# Long non‐coding RNA LOC100133669 promotes cell proliferation in oesophageal squamous cell carcinoma

**DOI:** 10.1111/cpr.12750

**Published:** 2020-03-04

**Authors:** Zhuzhu Guan, Yali Wang, Yu Wang, Xiaoxu Liu, Yan Wang, Weimin Zhang, Xinming Chi, Yan Dong, Xuefeng Liu, Shujuan Shao, Qimin Zhan

**Affiliations:** ^1^ Institute of Cancer Stem Cell Dalian Medical University Dalian China; ^2^ State Key Laboratory of Molecular Oncology National Cancer Center/National Clinical Research Center for Cancer/Cancer Hospital Chinese Academy of Medical Sciences and Peking Union Medical College Beijing China; ^3^ Key Laboratory of Carcinogenesis and Translational Research (Ministry of Education/Beijing) Laboratory of Molecular Oncology Peking University Cancer Hospital & Institute Beijing China; ^4^ Liaoning Key Laboratory of Proteomics Dalian Medical University Dalian China; ^5^ College of Stomatology Dalian Medical University Dalian China

**Keywords:** ESCC, lncRNA, LOC100133669, proliferation, Tim50

## Abstract

**Objectives:**

LOC100133669 is a lncRNA whose function during tumorigenesis remains unclear now. Thus, we aimed to explore its clinical significance and function in oesophageal squamous cell carcinoma (ESCC).

**Materials and Methods:**

ISH was used to detect LOC100133669 expression in ESCC tissues. The full‐length LOC100133669 was identified by using RACE assay. Subcellular distribution of LOC100133669 was examined by nuclear/cytoplasmic RNA fractionation and qPCR. The role of LOC100133669 in ESCC cell growth was determined by colony formation, MTT and flow cytometry experiments in vitro, as well as xenograft tumour experiment in vivo. RNA pull‐down assay was performed to find LOC100133669‐interacted protein, which was further examined by RIP, IP, Western blot and rescue experiments.

**Results:**

LOC100133669 was upregulated in ESCC tissues compared with adjacent non‐tumour tissues. High LOC100133669 expression was associated with poor prognosis of patients with ESCC. We defined LOC100133669 to be 831 nt in length and mainly localized in the cytoplasm of ESCC cells. Knockdown of LOC100133669 inhibited ESCC cell proliferation and cell cycle progression, while overexpression of LOC100133669 showed the opposite effects. Furthermore, LOC100133669 could bind to Tim50 and upregulated its protein level through inhibiting ubiquitination. Overexpression of Tim50 in part abolished the LOC100133669 depletion–caused inhibitory effect on ESCC cell proliferation.

**Conclusions:**

LOC100133669 plays an oncogenic role in ESCC and may serve as a promising diagnostic marker and therapeutic target for ESCC patients.

## 1 INTRODUCTION

Oesophageal carcinoma is one of the most lethal gastrointestinal tumours in the world, with the overall five‐year survival rate only of 15%‐25%.^1^ In 2018, 572 000 new cases and 509 000 deaths of oesophageal carcinoma were estimated worldwide.^2^ The distribution of oesophageal carcinoma varies with geography.^3^, ^4^ China is one of the countries with the highest incidence and mortality of oesophageal carcinoma.^3^ According to the histological classification, oesophageal carcinoma is mainly divided into oesophageal squamous cell carcinoma (ESCC) and oesophageal adenocarcinoma (EAC). ESCC counts for most oesophageal carcinoma in China,^5^ which may be related to diet and living habits, alcohol consumption and cigarette smoking, environmental and genetic factors.^6^, ^7^ Currently, since there is a lack of effective detection markers and treatment targets for patients with ESCC, the exploration of promising biomarkers and molecular mechanisms of ESCC is an urgent goal.

Long non‐coding RNAs (lncRNAs) are commonly defined as a class of transcripts that are over 200 nucleotides (nt) in length and cannot encode proteins.^8^ According to the location relative to other transcripts, lncRNAs are generally categorized as sense, antisense, bidirectional, intronic and intergenic ones.^9^ Recent studies have shown that lncRNAs participate in the regulation of gene expression at epigenetic,^10^ transcriptional^11^, ^12^ and post‐transcriptional^13^, ^14^ levels, performing as signals, scaffolds, guides and decoys.^15^ Even in the same cellular environment, a lncRNA regulates gene expression in various ways. For example, in the nucleus, CCAT1 acts as a scaffold binding both PRC2 and SUV39H1 to regulate the histone methylation of SPRY4 promoter; in the cytoplasm, CCAT1 serves as a molecular decoy of miR‐7 to affect HOXB13 expression.^16^

Disorder of lncRNAs is closely related to various diseases, including metabolic diseases,^17^, ^18^ neurological diseases,^19^, ^20^ autoimmune diseases^21^, ^22^ and particularly cancers.^23^, ^24^ A great number of lncRNAs are reported to be dysregulated in human cancers and play critical roles during carcinogenesis.^23^, ^24^ HOTAIR, a lncRNA localized on the HOXC locus regulating the expression of HOXD genes,^10^ is overexpressed and exhibits oncogenic activity in a variety of human cancers.^25^, ^26^, ^27^ PCAT‐1, a lncRNA originally identified to be overexpressed in prostate cancer,^28^ is also upregulated in other human cancers and contributes to cancer progression.^29^, ^30^ Although more and more researchers have shifted their focus of cancer research to lncRNAs, the roles and mechanisms of lncRNAs in human cancers, including ESCC, are still unclear.

In this study, we focused our attention on a NCBI RefSeq‐annotated lncRNA, LOC100133669 (RefSeq accession number: NR_026913.1), which is located on human chromosome 8q24.3. The function of LOC100133669 during tumorigenesis remains unclear now. Here, we aimed to study the clinical significance and function of LOC100133669 in ESCC and hoped it could be a new molecular target for the diagnosis and treatment of ESCC.

## 2 MATERIALS AND METHODS

### 2.1 Cell lines and cell culture

The human ESCC cell lines COLO680N, YES2, KYSE30, KYSE70, KYSE140, KYSE150, KYSE180, KYSE410, KYSE450 and KYSE510 were from Professor Yutaka Shimada of Kyoto University as gifts. The human embryonic kidney cell line HEK293T was obtained from American Type Culture Collection (ATCC, USA). ESCC cells were cultured in RPMI 1640 medium (Gibco), and HEK293T cells were cultured in Dulbecco's modified Eagle's medium (DMEM; Gibco). All culture medium was supplemented with 10% foetal bovine serum (FBS). Cells were maintained in a humidified incubator (Thermo Scientific) at 37°C with 5% CO_2_.

### 2.2 Plasmid construction, transfection and establishment of stable cell line with LOC100133669 overexpression

To construct the pcDNA3.1(+) vector expressing LOC100133669 (pcDNA3.1‐669), the full‐length LOC100133669 sequence was synthesized and cloned into pcDNA3.1(+) by Generay (Shanghai, China). pEGFP‐Tim50 vector was obtained by subcloning the Tim50 from pLX304‐Blast‐V5‐Tim50 into pEGFP‐N1 by Generay (Shanghai, China). The indicated cells were transfected with the plasmids using Lipofectamine 2000 (Invitrogen) following the manufacturer's instructions. KYSE450 cells transfected with pcDNA3.1(+) empty vector or pcDNA3.1‐669 vector were selected by G418 to generate control and LOC100133669‐stable overexpression cell lines.

### 2.3 Construction of shRNA lentiviral vector and establishment of stable cell line with LOC100133669 knockdown

Short hairpin RNA (shRNA) sequence targeting LOC100133669 was designed using the online design software (https://portals.broadinstitute.org/gpp/public/seq/search) and was cloned into pLKO.1 vector to generate the pLKO.1‐sh669. pLKO.1 empty vector or pLKO.1‐sh669 vector was transfected into HEK293T cells together with the packaging plasmids using Lipofectamine 2000 (Invitrogen) following the manufacturer's instructions. The lentiviruses in the medium were collected 72 hours later and then infected the KYSE510 cells in the presence of polybrene (Sigma). KYSE510 control and LOC100133669‐stable knockdown cell lines were obtained by puromycin screening. The shRNA sequences are as follows: forward 5′‐CCGGACTCAGGAGAGTGAAAGAAACCTCGAGGTTTCTTTCACTCTCCTGAGTTTTTTG‐3′, reverse 5′‐AATTCAAAAAACTCAGGAGAGTGAAAGAAACCTCGAGGTTTCTTTCACTCTCCTGAGT‐3′.

### 2.4 RNA interference

All small interfering RNAs (siRNAs) against LOC100133669 were purchased from Invitrogen (USA). The target sequences are as follows: si669‐1, sense 5′‐CCUGAACAAGAACUCAGGAGAGUGA‐3′, antisense 5′‐UCACUCUCCUGAGUUCUUGUUCAGG‐3′; and si669‐2, sense 5′‐ACACAUGGCUGGGUUAGUAGCUGAA‐3′, antisense 5′‐UUCAGCUACUAACCCAGCCAUGUGU‐3′. ESCC cells were transiently transfected with siRNAs using Lipofectamine 2000 (Invitrogen) following the manufacturer's instructions.

### 2.5 Tissue microarrays and RNA in situ hybridization (ISH)

The ESCC tissue microarrays with clinicopathological features and survival times were purchased from Shanghai Outdo Biotech Co., Ltd (SOBC). RNA ISH was performed to detect the expression of LOC100133669. Briefly, after dewaxing and digesting with protease K, ESCC tissue microarrays were hybridized with a double (5′ and 3′) digoxin‐labelled LNA probe specific for LOC100133669 (5′‐AGTGAGGCTGGAAGGCTGGAT‐3′; Exiqon) at 50°C for 1 hour and were subsequently incubated with alkaline phosphatase–conjugated digoxin antibody at 4°C overnight. LOC100133669 expression was visualized by 4‐nitroblue tetrazolium (NBT)/5‐bromo‐4‐chloro‐3‐indolyl phosphate (BCIP) substrate. Cell nuclei were counterstained with nuclear fast red.

The ISH results were evaluated by a semi‐quantitative scoring criterion based on the positive staining intensity (0, no positive staining; 1, weak positive staining; 2, intermediate positive staining; 3, strong positive staining) and the percentage of positive cells (0, no positive area; 1, 1%‐25%; 2, 26%‐50%; 3, 51%‐75%; 4, 76%‐100%). The comprehensive score = the positive staining intensity × the percentage of positive cells.

### 2.6 RNA extraction, reverse transcription‐quantitative PCR (RT‐qPCR) and semi‐quantitative PCR

Total RNA extracted from ESCC cells with TRIzol reagent (Invitrogen) was reverse‐transcribed to cDNA with oligo dT primer or random 6 mers using a PrimeScript™ RT Reagent Kit with gDNA Eraser (Takara, China). qPCR was performed using SYBR Premix Ex Taq (Takara, China) on a Stratagene Mx3000P qPCR System (Agilent Technologies). Each sample was analysed in triplicate. Semi‐quantitative PCR was performed using 2 × EasyTaq PCR SuperMix (TransGen Biotech, China) on a T100 Thermal Cycler (Bio‐Rad). The PCR products were analysed on an agarose gel. All procedures were carried out according to the manufacturer's protocols. GAPDH was used as reference. The primers are as follows: LOC100133669, forward 5′‐GCTGAAGCATCCTGAACAAGAAC‐3′, reverse 5′‐AGGACGCTTCGGTCTCTGTAC‐3′; myc, forward 5′‐TCTCCGTCCTCGGATTCTCT‐3′, reverse 5′‐TTCTTGTTCCTCCTCAGAGTCG‐3′; pre‐myc, forward 5′‐ACCCCTTTAACTCAAGACTGCC‐3′, reverse 5′‐CCGAGTCGTAGTCGAGGTCAT‐3′; GAPDH, forward 5′‐GCTGAGAACGGGAAGCTTGT‐3′, reverse 5′‐GCCAGGGGTGCTAAGCAGTT‐3′; and Tim50, forward 5‘‐CAGGAACCGTACTACCAGCC‐3’, reverse 5‘‐TGGGCGCTTCTTAAACCTCC‐3’.

### 2.7 Rapid amplification of cDNA ends (RACE)

The 5′‐ and 3′‐RACE experiments were performed using the SMARTer RACE Kit (Clontech) according to the manufacturer's instructions. The RACE PCR products were separated on an agarose gel. PCR bands were cloned into pEASY‐T1 vector (TransGen Biotech, China) and were sequenced. The RACE primers targeting LOC100133669 used for the nested PCR are listed below: 5′‐RACE outer primer, 5′‐TGGGTGATGGGGAGAGAGAGGACGCTTC‐3′; 5′‐RACE inner primer‐1, 5′‐CAACGAGGACTGTCCAGGTGTCAGTGAC‐3′; 5′‐RACE inner primer‐2, 5′‐GGTGAAGAAAGTTCCCAATGGCTGTGAT‐3′; 3′‐RACE outer primer, 5′‐GGCTCCATCCAGCCTTCCAGCCTCACTA‐3′; and 3′‐RACE inner primer, 5′‐AGGCCGTGGTCACACATGGCTGGGTTAG‐3′.

### 2.8 Nuclear/cytoplasmic RNA fractionation

Nuclear and cytoplasmic fractions were isolated from KYSE450 and KYSE510 cells using nucleoplasmic fractionation buffer (140 mmol/L NaCl, 1.5 mmol/L MgCl_2_, 10 mmol/L Tris‐HCl pH 8.5, 0.5% NP‐40). Briefly, cell pellet was resuspended with nucleoplasmic fractionation buffer and incubated on ice for 5 minutes. After centrifugation at 5000 × ***g*** for another 5 minutes, the supernatant and pellet were collected as the cytoplasmic and nuclear fractions, respectively. RNA was extracted from nuclear/cytoplasmic fractions, and RT‐qPCR was then used to evaluate the relative levels of LOC100133669, myc precursor RNA (pre‐myc) and GAPDH in each sample.

### 2.9 Colony formation assay

KYSE450 control and LOC100133669‐stable overexpression cells, KYSE510 control and LOC100133669‐stable knockdown cells, and KYSE150/KYSE510 cells transiently transfected with the control siRNA or siRNAs against LOC100133669 for 24 hours were trypsinized into a single‐cell suspension and seeded. Ten days later, the colonies were fixed with methanol, stained with crystal violet solution and photographed. Colonies containing more than 50 cells were counted.

### 2.10 MTT assay

KYSE450 control and LOC100133669‐stable overexpression cells, KYSE510 control and LOC100133669‐stable knockdown cells, and KYSE150/KYSE510 cells transiently transfected with the control siRNA or siRNAs against LOC100133669 for 24 hours were trypsinized into a single‐cell suspension, seeded and cultured for 6 days. 10 μL of MTT (5 mg/mL; Sigma) was added into each well daily. After incubation for 4 hours at 37°C, supernatant was removed and dimethyl sulfoxide (DMSO; Sigma) was added into each well. The viability was evaluated at a wavelength of 492 nm using a microplate reader (Sunrise; TECAN).

### 2.11 Cell cycle analysis

To synchronize ESCC cells at G2/M phase, KYSE450 control and LOC100133669‐stable overexpression cells, and KYSE150/KYSE510 cells transiently transfected with the control siRNA or siRNAs against LOC100133669 for 48 hours were treated with nocodazole (0.6 µg/mL) for 24 hours; to synchronize ESCC cells at G0/G1 phase, KYSE450 control and LOC100133669‐stable overexpression cells, and KYSE510 cells transiently transfected with the control siRNA or siRNAs against LOC100133669 for 24 hours were cultured without serum for 48 hours. Then, the blocked cells were released, collected at the indicated time points and fixed with ice‐cold 70% ethanol at −20°C overnight. The fixed cells were treated with RNase A and stained with propidium iodide (PI). Finally, the cells were analysed with BD Accuri C6 Flow Cytometer (BD Biosciences) equipped with ModFit LT software (Version 5.0).

### 2.12 RNA pull‐down assay

RNA pull‐down assay was performed as described previously.^31^ Briefly, template DNA for in vitro transcription of LOC100133669 was obtained by linearizing pcDNA3.1‐669 vector with restriction enzyme EcoRI at the 3′ end. Template DNA for in vitro transcription of GAPDH was PCR‐amplified using the primers containing T7 promoter sequence as follows: T7‐GAPDH, forward, 5′‐GATCACTAATACGACTCACTATAGGGAGAATGGGGAAGGTGAAGGTCG‐3′, reverse, 5′‐TTACTCCTTGGAGGCCATGTG‐3′. Biotin‐labelled RNAs of LOC100133669 and GAPDH were transcribed in vitro using the MEGAscript™ T7 Transcription Kit (Invitrogen) with biotin‐16‐UTP (Invitrogen). Cell extracts were incubated with RNAs for 30 minutes, followed by adding streptavidin agarose beads (Invitrogen) for further incubation. After washing for 5‐6 times, LOC100133669‐associated proteins, which were retrieved from beads, were subjected to SDS‐PAGE and silver staining. Differential protein bands were excised and identified by mass spectrometry.

### 2.13 Western blot assay and antibodies

Total proteins extracted from cells were separated by SDS‐PAGE and transferred to PVDF membranes. Then, the membranes were blocked with 5% non‐fat milk and subsequently incubated with primary antibodies against Tim50 (Proteintech Group, China) or β‐actin (Proteintech Group, China) at 4°C overnight. After incubation with the secondary antibody at room temperature for 1 hour, the bands were observed with the ECL kit and quantified by densitometry (Gel‐PRO Analyzer). β‐actin was used as reference.

### 2.14 RNA immunoprecipitation (RIP) assay

RIP assay was conducted with Magna RIP™ RNA‐Binding Protein Immunoprecipitation Kit (Millipore, USA) according to the manufacturer's instructions. Briefly, cells were lysated in lysis buffer containing protease inhibitor cocktail and RNase inhibitor. Then, cell extracts were incubated with magnetic beads conjugated with control IgG or anti‐Tim50 antibody (Proteintech Group, China). Immunoprecipitated RNAs were purified and quantified by RT‐qPCR.

### 2.15 Immunoprecipitation (IP) assay

KYSE510 control cells and LOC100133669‐stable knockdown cells were treated with MG132 (20 μmol/L) for 8 hours to inhibit proteasome degradation. Then, treated cells were lysated with lysis buffer containing a protease inhibitor cocktail. Cell extracts were incubated with control IgG or anti‐Tim50 antibody. Magnetic beads were then added and incubated. After washing, precipitated proteins were detected by Western blot with an antibody against ubiquitin (Proteintech Group, China).

### 2.16 In vivo xenograft tumour experiment

In vivo xenograft tumour experiment was performed as described previously.^32^ KYSE510 control cells and LOC100133669‐stable knockdown cells were subcutaneously injected into the left and right flanks of 6‐week‐old NU/NU nude mice (Charles River, China), respectively. At 30 days after inoculation, mice were sacrificed and tumour weights were measured. The experiment protocol was approved by the Animal Care and Use Committee of Dalian Medical University.

### 2.17 Statistical analysis

All statistical analyses were performed using SPSS19.0 software or GraphPad Prism 5.0 software. Data are expressed as mean ± standard deviation (SD). One‐way analysis of variance (ANOVA) was used for multigroup comparison, and Student's *t* test was used for comparison between two groups. Overall survival was analysed by the Kaplan‐Meier method, and comparison was performed using log‐rank test. Prognosis factors were analysed by Cox proportional hazard regression model. *P* < .05 was considered statistically significant.

## 3 RESULTS

### 3.1 LOC100133669 is upregulated in ESCC tissues and correlates with poor prognosis

**Figure 1:**
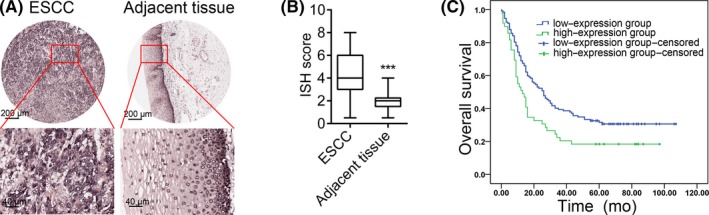
LOC100133669 expression is elevated in ESCC tissues and is associated with poor prognosis. A, Representative images of ISH for LOC100133669 in ESCC tissue microarrays. B, Histogram analysis of LOC100133669 expression in matched ESCC tissues and adjacent non‐tumour tissues (n = 155). Statistical significance was evaluated using paired Student's *t* test, ***P < .001. C, Kaplan‐Meier curve showing overall survival of ESCC patients stratified by LOC100133669 level. Statistical significance was evaluated using log‐rank test (*χ* ^2^ = 6.296, *P* = .012)

To investigate the clinical significance of LOC100133669 in ESCC, we detected the expression of LOC100133669 by RNA ISH in tissue microarrays, containing matched 155 human ESCC tissues and adjacent non‐tumour tissues, as well as other unmatched 26 human ESCC tissues. The results showed that LOC100133669 was mainly localized in the cytoplasm and was highly expressed in ESCC tissues compared with adjacent non‐tumour tissues (Figure 1A,B). We then divided the 181 ESCC tissues into high–LOC100133669 expression group (n = 49, ISH score ≥ 6) and low–LOC100133669 expression group (n = 132, ISH score < 6). High LOC100133669 expression was not associated with clinicopathological features of ESCC patients (Table S1). However, the patients with high LOC100133669 expression had shorter overall survival time than those with low LOC100133669 expression, as analysed by the Kaplan‐Meier method (log‐rank test, *χ* ^2^ = 6.296, *P* = .012; Figure 1C), indicating that LOC100133669 overexpression was associated with poor survival of ESCC patients.

Moreover, to test whether LOC100133669 can serve as an independent prognostic factor for the overall survival of ESCC patients, LOC100133669 expression (ISH score < 6 vs ≥6), gender (female vs male), age (≤65 years vs >65 years), pathological grades (grade I vs II‐III), tumour size (≤5 cm vs >5 cm), primary lesion infiltration (T1‐T2 vs T3), lymph node metastasis (N0 vs N1‐N3) and AJCC stages (stage I‐II vs III) were taken as dependent variables. Multivariate Cox regression analysis showed that LOC100133669 score ≥ 6, male, tumour size > 5 cm and AJCC stages III were poor prognosis risk factors for ESCC patients (Table 1). The adverse prognosis risk of ESCC patients with high LOC100133669 expression was 2.009 times higher than that of those with low LOC100133669 expression (95% CI 1.340‐3.010, *P* = .001).

**Table 1:** Multivariate Cox analysis

Variable	*P*	HR	95% CI	
			Lower	Upper
Expression of LOC100133669	.001	2.009	1.340	3.010
Gender	.025	1.838	1.080	3.126
Age	.128	1.346	0.918	1.972
Pathological grades	.850	1.040	0.692	1.563
Tumour size	.008	1.701	1.146	2.527
Primary lesion infiltration (T)	.299	1.358	0.762	2.418
Lymph node metastasis (N)	.262	0.500	0.149	1.678
AJCC stages	.037	3.787	1.083	13.236

### 3.2 Identification and characterization of LOC100133669

**Figure 2:**
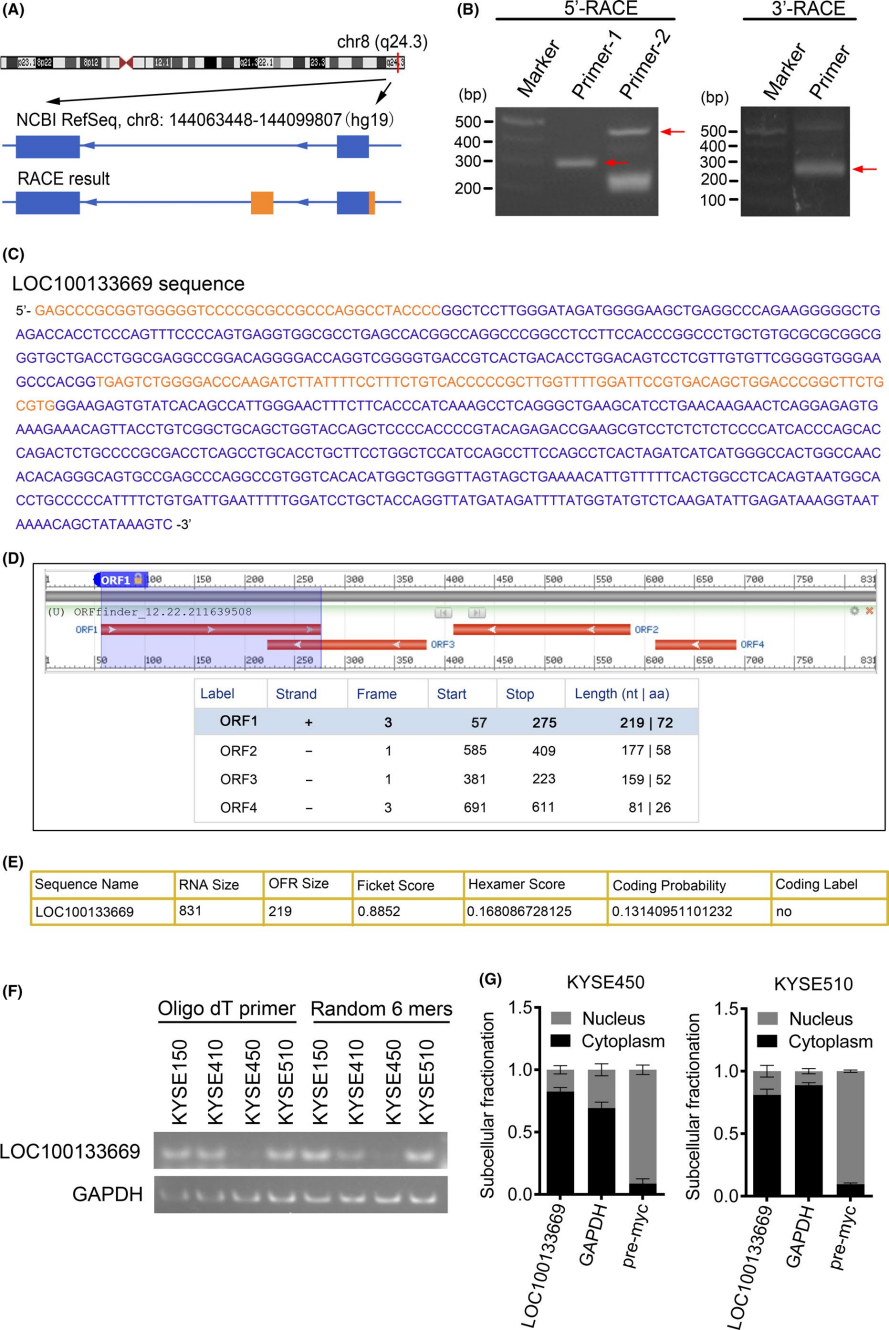
Identification and characterization of LOC100133669. A, Schematic diagram of LOC100133669 from NCBI RefSeq and RACE. Blue boxes, exons annotated by NCBI RefSeq. Orange boxes, additional sequence and exon determined by RACE. Blue lines, introns. Arrows on blue lines indicate transcriptional directions. B, Gel electrophoresis of nested PCR products from 5′‐RACE and 3′‐RACE. Arrows indicate purpose strips. C, The full‐length sequence of LOC100133669 transcript. Blue, reference sequence from NCBI RefSeq. Orange, added sequence from RACE. D, E, ORF finder software (D) and CPAT software (E) prediction for protein‐coding potential of LOC100133669. F, Gel electrophoresis of PCR products reversed with oligo dT primer or random 6 mers, respectively, in KYSE150, KYSE410, KYSE450 and KYSE510 cells. G, Subcellular distribution of LOC100133669 in KYSE450 and KYSE510 cells. GAPDH and myc precursor RNA (pre‐myc) were served as cytoplasmic and nuclear references, respectively. Data are presented as mean ± SD, n = 3

The UCSC Genome Browser (http://genome.ucsc.edu) shows that LOC100133669 is located on the human chromosome 8q24.3 and composed of two exons annotated by NCBI RefSeq (exon 1, 231 nt; exon 2, 471 nt) (Figure 2A). To identify the full‐length of LOC100133669 transcript in the ESCC cells, RACE assay was performed, and the PCR products of 5′‐ and 3′‐RACE are shown in Figure 2B. Based on the results from Sanger sequencing, the full length of LOC100133669 transcript was identified to be 831 nt, with an additional 41 nt at the 5′ end of exon 1 and an additional exon (88 nt) between exons 1 and 2 (Figure 2A and 2C). ORF finder from the NCBI (https://www.ncbi.nlm.nih.gov/orffinder/) failed to predict a protein of LOC100133669 sequence as determined by RACE (Figure 2D). CPAT software^33^ (http://lilab.research.bcm.edu/cpat/index.php) also showed that LOC100133669 had limited protein‐coding potential (Figure 2E). In addition, in the 3′‐RACE experiment, we found that LOC100133669 had a poly(A) tail. Consistently, the PCR products of LOC100133669, amplified from cDNA reversed with oligo dT primer or random 6 mers, respectively, were basically the same (Figure 2F), indicating that LOC100133669 is a lncRNA with a poly(A) tail. The localization of lncRNAs in cells is closely related to their functions and mechanisms.^34^ Thus, we detected the distribution of LOC100133669 in the nucleus and cytoplasm of KYSE450 and KYSE510 cells by subcellular fractionation and RT‐qPCR. The results showed that LOC100133669 was mainly localized in the cytoplasm of ESCC cells (Figure 2G), which is consistent with the results from ISH.

### 3.3 LOC100133669 promotes ESCC cell proliferation

**Figure 3:**
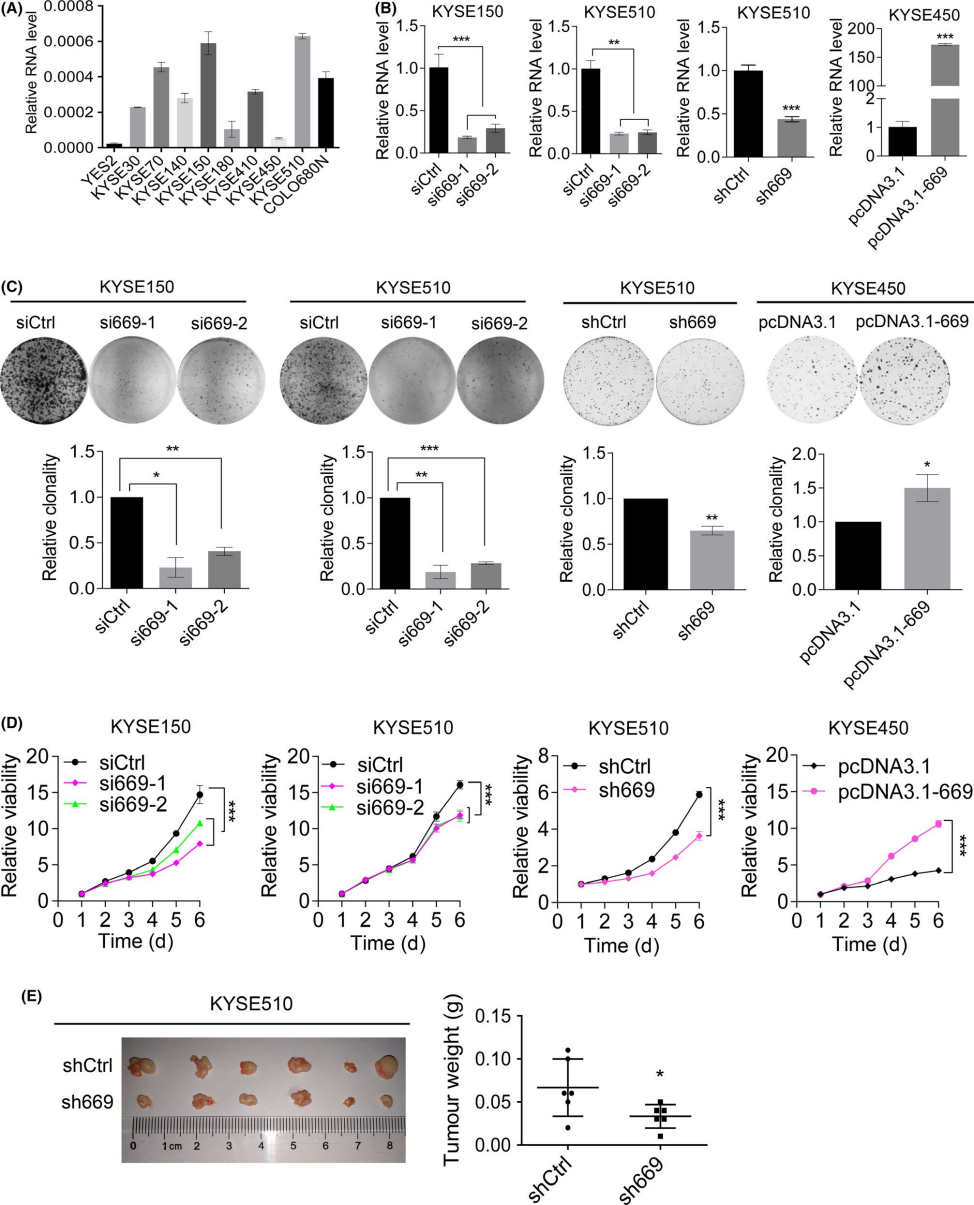
LOC100133669 promotes ESCC cell proliferation. A, RT‐qPCR analysis of LOC100133669 expression in different ESCC cell lines. B, RT‐qPCR analysis of LOC100133669 expression in KYSE150 and KYSE510 cells transiently transfected with control siRNA (siCtrl) or siRNAs targeting LOC100133669 (si669‐1 and si669‐2) for 48 h, as well as in KYSE510 control cells (shCtrl) and LOC100133669‐stable knockdown cells (sh669), and in KYSE450 control cells (pcDNA3.1) and LOC100133669‐stable overexpression cells (pcDNA3.1‐669). C, D, Colony formation (C) and MTT (D) assays were performed in cells as shown in B. E, KYSE510 control cells and LOC100133669‐stable knockdown cells were injected subcutaneously into nude mice. The established tumours and tumour weights are shown (n = 6). Data are presented as mean ± SD, n = 3, unless otherwise specified. *P < .05, **P < .01, ***P < .001

To study whether LOC100133669 plays roles in ESCC tumorigenesis, we first detected the LOC100133669 expression in ten ESCC cell lines by RT‐qPCR (Figure 3A). Then, KYSE150 and KYSE510 cells that showed high level of LOC100133669 were transfected with two siRNAs to reduce LOC100133669 expression (Figure 3B). We also established LOC100133669‐stable knockdown and control cell lines in KYSE510 cells with lentivirus‐delivered shRNA (Figure 3B). Subsequently, KYSE450 cells with relatively low level of LOC100133669 were transfected with an overexpression vector (pcDNA3.1‐669) to establish the cell line stably expressing LOC100133669 (Figure 3B). Colony formation assay was then performed to investigate the effect of LOC100133669 on ESCC cell proliferation. The results showed that knockdown of LOC100133669 significantly reduced the colony formation capacity of ESCC cells, while overexpression of LOC100133669 had the converse effect (Figure 3C). Consistent results were obtained from MTT assay (Figure 3D). The tumorigenic role of LOC100133669 was further investigated using xenograft tumour experiment in vivo. KYSE510 control cells and LOC100133669‐stable knockdown cells were injected subcutaneously into nude mice. As shown in Figure 3E, knockdown of LOC100133669 reduced the ability of KYSE510 cells to form tumours in nude mice. Collectively, these data indicate that LOC100133669 can promote the proliferation of ESCC cells.

### 3.4 Effect of LOC100133669 on cell cycle progression of ESCC cells

**Figure 4:**
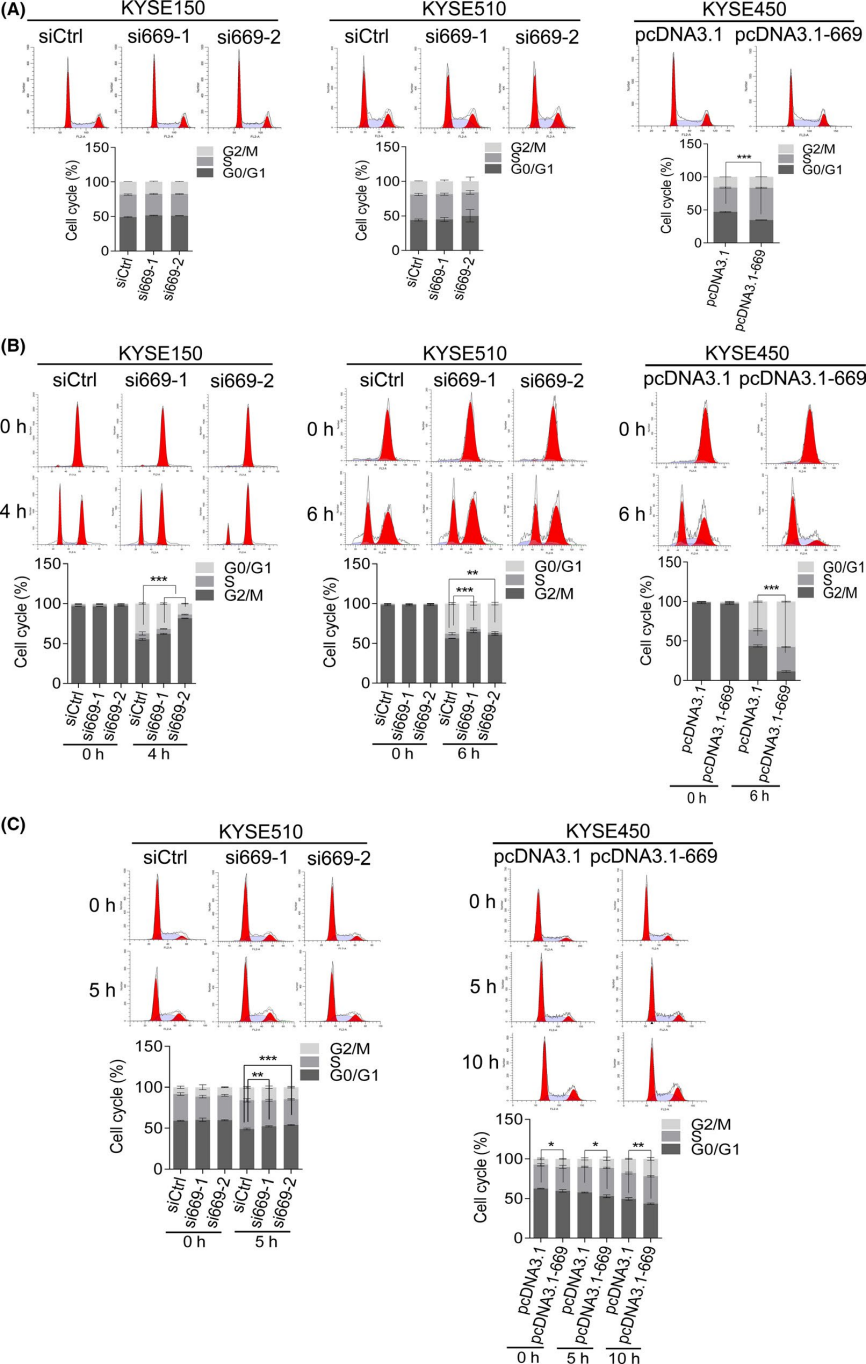
LOC100133669 promotes ESCC cell cycle progression. A, Flow cytometry analysis of cell cycle distribution in KYSE150 and KYSE510 cells transiently transfected with control siRNA or siRNAs targeting LOC100133669 for 48 h, as well as in KYSE450 control cells and LOC100133669‐stable overexpression cells. B, C, The ESCC cells as indicated were synchronized with nocodazole for 24 h (B) or serum starvation for 48 h (C), respectively. Then, the blocked cells were recovered and collected at the indicated time points. The cell cycle distribution was detected by PI staining, followed by flow cytometry. The difference of G2/M phase (for nocodazole treatment) or G0/G1 phase (for serum starvation) was analysed. Data are presented as mean ± SD, n = 3. *P < .05, **P < .01, ***P < .001

As cell cycle progression plays an important role in determining cell growth, we then aimed to explore whether LOC100133669 contributes to cell cycle progression of ESCC cells. We found that the cell cycle distribution was not significantly changed in KYSE150 and KYSE510 cells upon depletion of LOC100133669, as determined by flow cytometric analysis (Figure 4A). However, a decrease in G0/G1 phase and an increase in S phase were observed in KYSE450 cells stably expressing LOC100133669 compared with control cells (Figure 4A). To further study the role of LOC100133669 in cell cycle progression, ESCC cells were synchronized at G2/M phase or G0/G1 phase with nocodazole treatment or serum starvation, respectively. Then, the blocked cells were released and cell cycle distribution was analysed at the indicated time points. After release from G2/M phase caused by nocodazole, knockdown of LOC100133669 led to an arrest at the G2/M phase of KYSE150 and KYSE510 cells when compared with control siRNA (Figure 4B). On the other hand, nearly all KYSE450 cells stably expressing LOC100133669 progressed through G2/M phase and entered G0/G1 phase, while many of the control cells were still present in G2/M phase (Figure 4B), indicating that LOC100133669 accelerates the process of ESCC cells from G2/M phase to G0/G1 phase. Consistently, after release from G0/G1 phase caused by serum starvation, LOC100133669‐depleted KYSE510 cells exhibited an arrest at the G0/G1 phase compared with control cells, while KYSE450 cells stably expressing LOC100133669 showed an opposite effect (Figure 4C), indicating that LOC100133669 could also promote the process of ESCC cells from G0/G1 phase to S phase.

**Figure 5:**
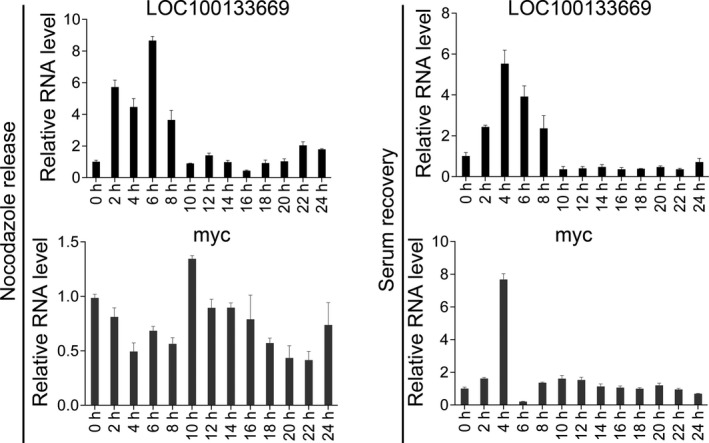
The expression of LOC100133669 after release from synchronization. KYSE510 cells were treated with nocodazole for 24 h or serum starvation for 48 h, respectively. Then, cells were recovered and collected every 2 h. The expression of LOC100133669 was detected by RT‐qPCR. Data are presented as mean ± SD, n = 3

Based on the effects of LOC100133669 on both G0/G1 and G2/M phases, we explored whether the expression of LOC100133669 is regulated by cell cycle progression. After synchronization at G0/G1 phase or G2/M phase with serum starvation or nocodazole treatment, respectively, KYSE510 cells were released and collected every 2 hours. RT‐qPCR was then used to detect the expression of LOC100133669, and the results showed that LOC100133669 expression was increased significantly at 2‐8 hours after re‐entering the cell cycle from both G0/G1 and G2/M phases (Figure 5). However, myc expression was only remarkably increased after release from serum starvation (Figure 5).

### 3.5 LOC100133669 interacts with Tim50

**Figure 6:**
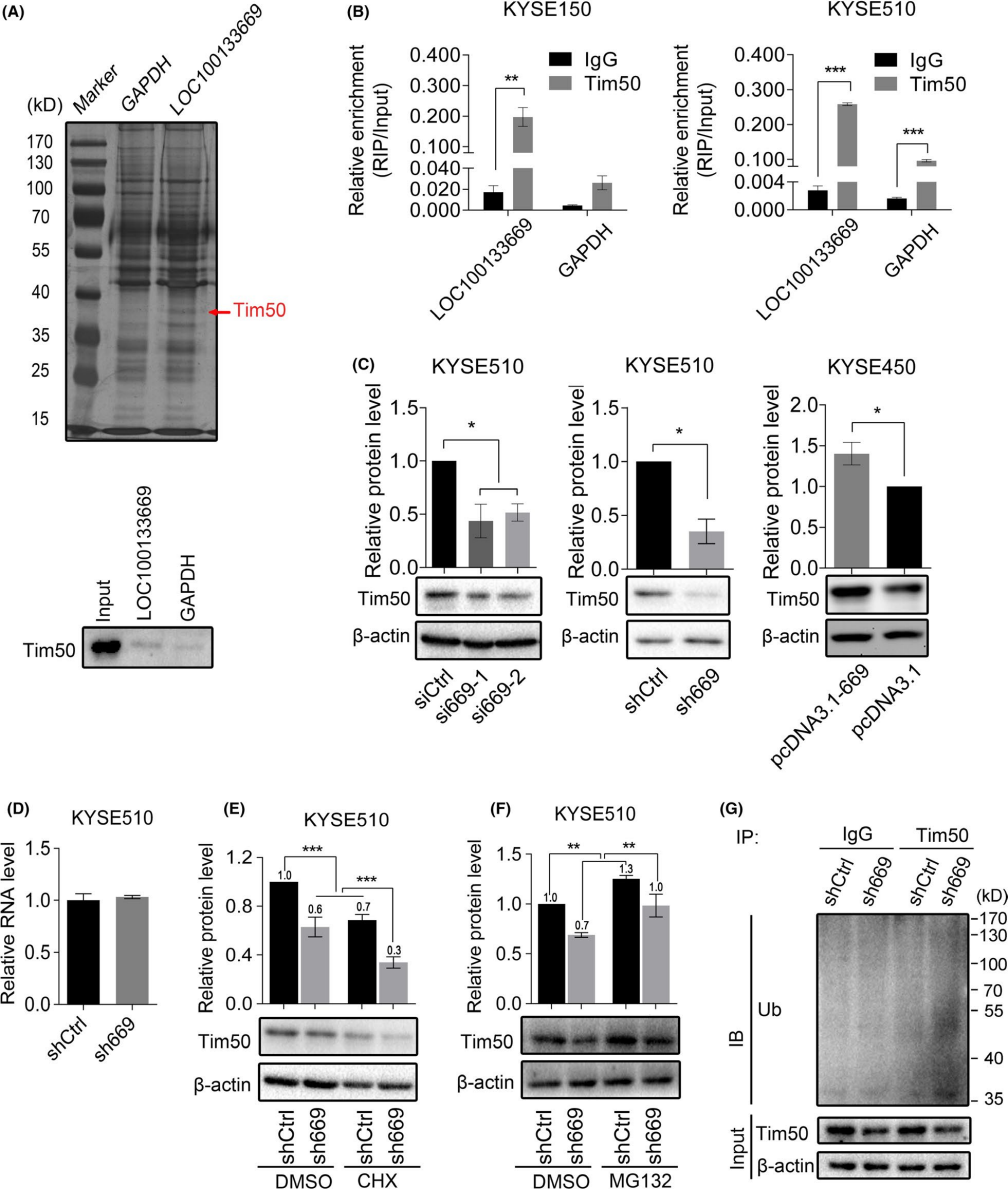
LOC100133669 binds to Tim50 and regulates its protein level via ubiquitination. A, Sliver staining of LOC100133669‐associated proteins following RNA pull‐down assay (up panel). Tim50, included in the differential band indicated by the red arrow, was identified as one of the LOC100133669‐associated proteins by mass spectrometry analysis. Western blot was performed to validate this interaction using anti‐Tim50 antibody (bottom panel). GAPDH was used as a negative RNA control. B, RIP assay confirming the interaction between LOC100133669 and Tim50 in KYSE150 (left panel) and KYSE510 (right panel) cells. C, Western blot analysis of Tim50 protein level in KYSE510 cells transiently transfected with control siRNA or siRNAs targeting LOC100133669 for 48 h, as well as in KYSE510 control cells and LOC100133669‐stable knockdown cells, and in KYSE450 control cells and LOC100133669‐stable overexpression cells. D, RT‐qPCR analysis of Tim50 mRNA level in KYSE510 control cells and LOC100133669‐stable knockdown cells. E, F, Western blot analysis of Tim50 protein level in KYSE510 control cells and LOC100133669‐stable knockdown cells, which were treated with CHX (100 μg/mL) (E) or MG132 (20 μmol/L) (F) for 8 h. G, IP coupled with Western blot analysis of Tim50‐associated ubiquitination in KYSE510 control cells and LOC100133669‐stable knockdown cells. Ub, ubiquitin. Data are presented as mean ± SD, n = 3. *P < .05, **P < .01, ***P < .001

Our next goal was to explore the underling mechanisms by which LOC100133669 plays an oncogenic role in ESCC. lncRNAs have been reported to interact with proteins to perform their functions.^10^, ^12^ Thus, we speculated that LOC100133669 may function via the same way. To search for LOC100133669‐associated proteins, we performed RNA pull‐down assay, followed by SDS‐PAGE analysis and silver staining (Figure 6A). Differential bands between LOC100133669 and negative control (GAPDH) were excised and subjected to mass spectrometry. One of the LOC100133669‐associated proteins was identified as translocase of inner mitochondrial membrane 50 (Tim50), which attracted our attention. Tim50, an important subunit of mitochondrial translocase complex TIM23, plays a critical role in transporting precursor protein into mitochondria^35^, ^36^, ^37^ and has been reported to be involved in tumorigenesis.^38^, ^39^ The interaction between LOC100133669 and Tim50 was validated by Western blot analysis (Figure 6A). To further confirm the association of LOC100133669 with Tim50, RIP assay was conducted and the results demonstrated the specific interaction between LOC100133669 and Tim50 in both KYSE150 and KYSE510 cells (Figure 6B).

### 3.6 LOC100133669 regulates Tim50 protein level via ubiquitination

Next, we examined whether LOC100133669 affects the protein level of Tim50. Western blot showed that Tim50 protein level was downregulated in LOC100133669‐knockdown cells and was upregulated in LOC100133669‐overexpression cells, when compared with corresponding control cells (Figure 6C). However, knockdown of LOC100133669 had no effect on the mRNA level of Tim50 (Figure 6D), indicating that LOC100133669 might regulate Tim50 at protein level. Some lncRNAs are reported to bind to proteins and affect their stability.^40^, ^41^ Hence, we treated KYSE510 control cells and LOC100133669‐stable knockdown cells with protein synthesis inhibitor cycloheximide (CHX) for 8 hours and subsequently detected the remaining Tim50 by Western blot. The results showed that the portion of remaining Tim50 in LOC100133669‐knockdown cells was lower than that in control cells (Figure 6E), indicating that Tim50 protein becomes less stable as a result of LOC100133669 knockdown. We then treated the cells with MG132 to block the proteasome degradation. Western blot showed that MG132 treatment resulted in an increased Tim50 protein level in both LOC100133669‐knockdown cells and control cells, but the increased ratio of Tim50 in LOC100133669‐knockdown cells was higher than that in control cells (Figure 6F), suggesting that proteasome pathway at least partially contributes to LOC100133669 knockdown–induced Tim50 protein degradation. To further confirm this observation, we performed IP assay in MG132‐treated cells with an antibody against Tim50, followed by Western blot using an anti‐ubiquitin antibody. Indeed, the level of Tim50 ubiquitination in LOC100133669‐knockdown cells was higher than that in control cells (Figure 6G). These data collectively indicate that LOC100133669 binds to Tim50 and upregulates its protein level through inhibiting ubiquitination.

### 3.7 Tim50 participates in the effect of LOC100133669 on ESCC cell proliferation

**Figure 7:**
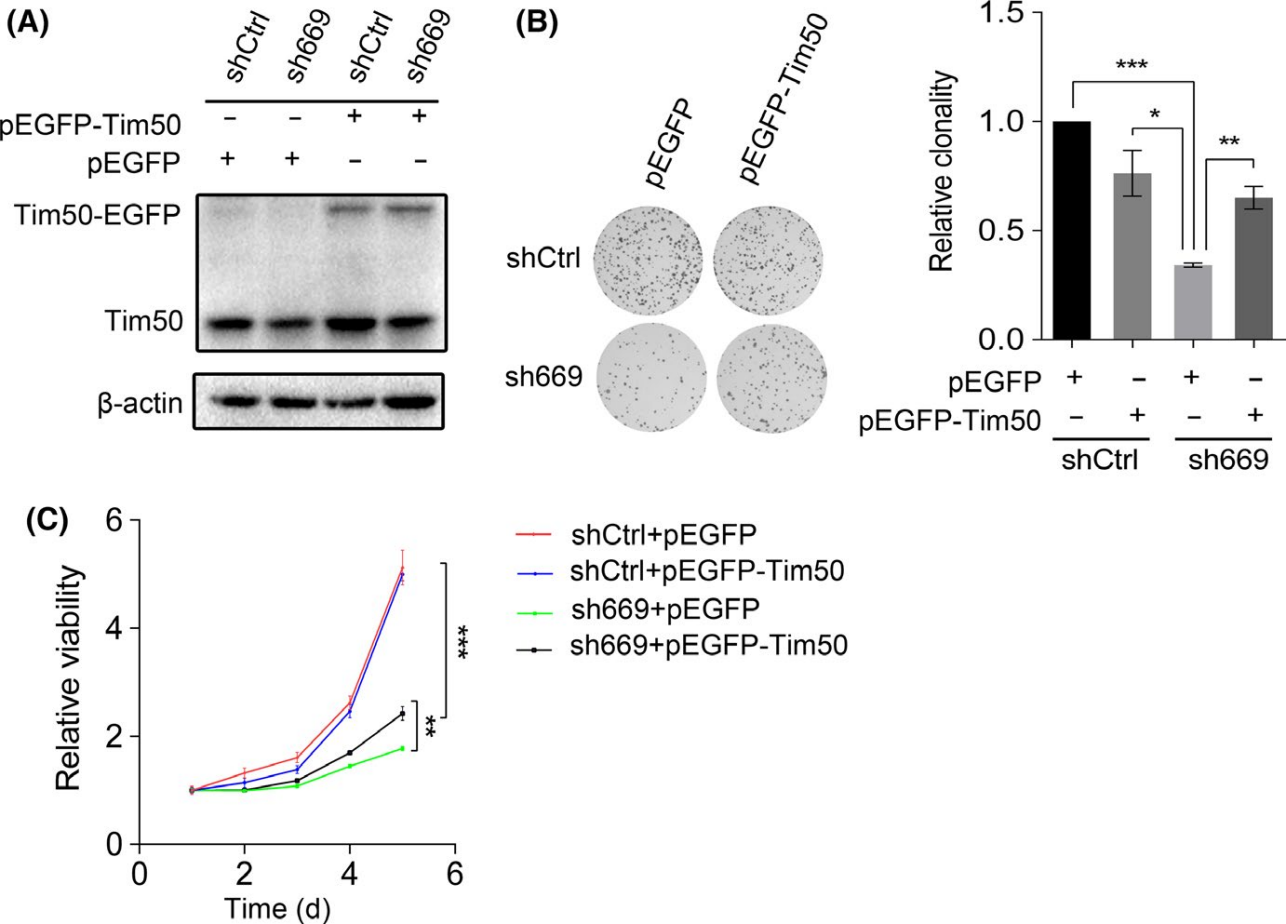
Tim50 overexpression partially compromised the effect of LOC100133669 knockdown on ESCC cell proliferation. A, Western blot analysis of Tim50 protein level in KYSE510 control cells and LOC100133669‐stable knockdown cells transiently transfected with pEGFP empty vector or pEGFP‐Tim50 vector for 48 h. B, C, Colony formation (B) and MTT (C) assays were performed in cells as shown in A. Data are presented as mean ± SD, n = 3. *P < .05, **P < .01, ***P < .001

To investigate whether Tim50 is involved in the LOC100133669‐regulated ESCC cell proliferation, rescue experiments were performed. We transfected Tim50 to KYSE510 control cells and LOC100133669‐stable knockdown cells (Figure 7A), followed by colony formation and MTT assays. The results revealed that Tim50 overexpression partially rescued LOC100133669 knockdown–induced decrease in ESCC cell proliferation (Figure 7B,C).

## 4 DISCUSSION

In recent years, lncRNAs have become a hotspot in cancer research. A large number of cancer‐associated lncRNAs are screened out using the subtractive hybridization method and especially high‐throughput chip or sequencing technology.^28^, ^42^ For example, MALAT‐1, a well‐known lncRNA, was identified from differential expression genes between primary non–small‐cell lung tumours that metastasized or did not subsequently.^43^ MALAT‐1 expression was also higher in ESCC tissues than that in adjacent normal tissues and was associated with poor prognosis.^44^, ^45^ At present, there is a lack of effective biomarkers for clinical diagnosis of ESCC. LncRNAs may provide a new area to find indicators for ESCC diagnosis. A study showed that HOTAIR in serum of patients with ESCC was significantly higher than that of controls and was decreased after operation, indicating that the expression of HOTAIR in serum may be a potential biomarker for the ESCC diagnosis.^46^

In this study, we focused our attention on a functionally unknown lncRNA, LOC100133669. We evidenced that LOC100133669 was upregulated in ESCC tissues, and served as a poor prognosis factor for ESCC patients. These results give us the impetus to further study the function and mechanism of LOC100133669 in ESCC. To do this, we first defined the full‐length of LOC100133669 transcript in ESCC cells using RACE assay, which was longer than the transcript annotated by NCBI RefSeq. Most lncRNAs are transcribed under the action of RNA polymerase II and have poly(A) tails, but do not encode proteins because of the lack of ORF.^8^, ^15^ Indeed, LOC100133669 was here identified as a poly(A)‐positive lncRNA with no protein‐coding potential.

Our functional experiments showed that LOC100133669 promoted the proliferation of ESCC cells. As we know, cell cycle progression is a key role in determining cell growth. Many lncRNAs have been demonstrated to be involved in cell cycle regulation. lncRNA DANCR was evidenced to be a direct MYC target that suppresses p21 expression to promote cell proliferation.^47^ lncRNA BLACAT1 was found to play a role in G0/G1 arrest by interacting with EZH2 to inhibit p15 expression.^48^ In our study, LOC100133669 was shown to accelerate the cell cycle procession of ESCC cells both from G2/M phase to G0/G1 phase and from G0/G1 phase to S phase. To explore whether the expression of LOC100133669 was cell cycle–regulated, KYSE510 cells were synchronized at G0/G1 phase or G2/M phase, respectively, by serum starvation or nocodazole treatment, and then released. Interestingly, no matter how the cells were blocked, LOC100133669 expression was increased significantly at 2‐8 hours after re‐entering the cell cycle. We speculate that LOC100133669 may not act on specific checkpoints of cell cycle, but accelerate the whole cell cycle process, thereby affecting cell proliferation.

ISH results showed that LOC100133669 was mainly localized in the cytoplasm, which is in accordance with the finding from nuclear and cytoplasmic RNA isolation, suggesting that LOC100133669 is likely to interact with molecules in the cytoplasm. Tim50, one of the LOC100133669‐associated proteins determined by RNA pull‐down assay, attracted our attention. Tim50 is a subunit of the TIM23 complex and is essential for directing translocation of preproteins into mitochondria.^35^, ^36^, ^37^ Mitochondria are important energy metabolism organelles, which participate in energy production, metabolism, cell death, cell signalling and oxidative stress.^49^ Increasing evidence has shown that mitochondrial dysfunction is closely related to tumorigenesis.^49^ Lack of Tim50 has been reported to lead to TIM23 complex and mitochondrial dysfunction.^50^ Furthermore, downregulation of Tim50 expression inhibited the growth and chemoresistance of human lung and breast cancer cells harbouring mutant p53.^38^ More recently, Tim50 was reported to facilitate NSCLC cell proliferation and invasion via ERK signalling pathway.^39^ In our study, knockdown of LOC100133669 downregulated the protein level of Tim50 through promoting its ubiquitination. Cytoplasmic lncRNAs have been reported to participate in regulating protein stability via ubiquitination.^40^, ^41^ For example, an oncogenic lncRNA GLCC1 was proven to stabilize transcriptional factor c‐Myc from ubiquitination degradation through directly binding to HSP90 chaperon.^51^ Moreover, we found that Tim50 overexpression partially abolished the reduction of ESCC cell proliferation caused by LOC100133669 knockdown. Our study suggests that LOC100133669 might change the expression level of Tim50, thereby affecting the function of mitochondria and then regulating cell growth.

In summary, LOC100133669 is highly expressed in ESCC tissues and has an association with patient prognosis. LOC100133669 promotes ESCC cell proliferation and cell cycle progression. LOC100133669 binds to Tim50 and upregulates its protein level through inhibiting ubiquitination. Tim50 overexpression partially rescues slowed ESCC cell growth due to LOC100133669 knockdown. Our findings identify LOC100133669 as a novel oncogene in ESCC, which may have the potential to be a target for ESCC diagnosis and therapy.

## CONFLICT OF INTEREST

None.

## AUTHOR CONTRIBUTIONS

Qimin Zhan, Shujuan Shao, Xuefeng Liu, Zhuzhu Guan, Yali Wang, Yan Wang, Weimin Zhang and Yan Dong participated in project design; Zhuzhu Guan, Yali Wang, Yu Wang, Xiaoxu Liu and Xinming Chi performed experiments and collected the data; Zhuzhu Guan was responsible for article writing; all authors gave advice to this manuscript; and Qimin Zhan, Shujuan Shao and Xuefeng Liu critically revised and approved the manuscript.

## Data Availability

The data that support the findings of this study are available from the corresponding author upon reasonable request.
