# Supplementary material for: Long non‐coding RNA LOC100133669 promotes cell proliferation in oesophageal squamous cell carcinoma
Source: Cell Prolif. 2020 Mar 4;53(4):e12750. doi: 10.1111/cpr.12750 (PMC7162797; doi:10.1111/cpr.12750)
Supplement: Supplementary file 1 [file CPR-53-e12750-s001.docx]

**TABLE S1** Analysis of clinicopathological features of LOC100133669 expression

| Clinicopathological  features | Low  expression  (n=132) | High  expression  (n=49) | X^2^ | P |
| --- | --- | --- | --- | --- |
| Gender |  |  | 0.000 | 0.995 |
| Male | 105 | 39 |  |  |
| Female | 27 | 10 |  |  |
| Age (year) |  |  | 0.058 | 0.810 |
| ≤ 65 | 70 | 25 |  |  |
| > 65 | 62 | 24 |  |  |
| Pathological grades |  |  | 1.883 | 0.170 |
| Ⅰ | 58 | 16 |  |  |
| Ⅱ-Ⅲ | 74 | 33 |  |  |
| Tumor sizes (cm) |  |  | 0.064 | 0.800 |
| ≤ 5 | 81 | 33 |  |  |
| > 5 | 43 | 16 |  |  |
| Primary lesion infiltration (T) |  |  | 0.002 | 0.965 |
| T1-T2 | 26 | 10 |  |  |
| T3 | 97 | 38 |  |  |
| Lymph node metastasis (N) |  |  | 0.184 | 0.668 |
| N0 | 64 | 22 |  |  |
| N1-N3 | 68 | 27 |  |  |
| Distant metastasis of tumor (M) |  |  | --- | --- |
| M0 | 132 | 49 |  |  |
| M1 | 0 | 0 |  |  |
| AJCC stages |  |  | 0.109 | 0.741 |
| Ⅰ-Ⅱ | 66 | 24 |  |  |
| Ⅲ | 59 | 24 |  |  |

8 cases of tumor size parameter were missing; 10 cases of primary lesion infiltration (T) parameter were missing; 8 cases of AJCC stages parameter were missing.
